# LKB1 expression and the prognosis of lung cancer: A meta-analysis

**DOI:** 10.1097/MD.0000000000027841

**Published:** 2021-11-19

**Authors:** Chunxuan Lin, Xiaochun Lin, Kunpeng Lin, Jialiang Tan, Chenggong Wei, Taisheng Liu

**Affiliations:** a Department of Respiratory Medicine, Guangdong Provincial Hospital of Integrated Traditional Chinese and Western Medicine, Foshan, Guangdong, P.R. China; b Department of Medical Examination Center, Guangzhou First People's Hospital, School of Medicine, South China University of Technology, Guangzhou, Guangdong, P.R. China; c Department of Abdominal Oncosurgery, Affiliated Cancer Hospital & Institute of Guangzhou Medical University, Guangzhou, Guangdong, P.R. China.

**Keywords:** LKB1, lung cancer, meta-analysis, prognosis

## Abstract

**Background:**

In the past few decades, many lines of evidence implicate the importance of liver kinase B1 (LKB1) as a tumor suppressor gene in the development and progression of solid tumours. However, the prognostic and clinicopathological value of LKB1 in patients with lung cancer are controversial. This article aimed to investigate the latest evidence on this question.

**Methods:**

A systematic literature searched in the PubMed, Web of Science, Embase, Cochrane library, Scopus until September 20, 2020. The association between overall survival (OS), relapse-free survival (RFS), progression-free survival (PFS), clinicopathological features and LKB1 were analysed by meta-analysis.

**Results:**

Eleven studies including 1507 patients were included in this meta-analysis. The pooled results revealed that low LKB1 expression was significantly associated with poor overall survival (OS) (HR = 1.67, 95% CI: 1.07–2.60, *P* = .024) in lung cancer. However, no association was found between LKB1 expression and DFS/PFS (HR = 1.29, 95% CI: 0.70–2.39, *P* = .410). Pooled results showed that low LKB1 expression was associated with histological differentiation (poor vs moderate or well, OR = 4.135, 95% CI:2.524–6.774, *P* < .001), nodal metastasis (absent vs present, OR = 0.503, 95% CI: 0.303–0.835, *P* = .008) and smoking (yes vs no, OR = 1.765, 95% CI: 1.120–2.782, *P* = .014).

**Conclusion:**

These results suggest that low expression of LKB1 can be considered as a unfavorable prognostic biomarker for human lung cancer, which should be further researched.

## 1 Introduction

Lung cancer is the most common cause of cancer-related deaths all over the world.^[1,2]^ About 1.8 million people are diagnosed with lung cancer every year, and 1.6 million people die because of this disease. Now several types of lung cancers can be recognized, such as small cell lung carcinomas, large cell carcinomas, adenocarcinomas, adenosquamous carcinomas and so on.^[3]^ Despite recent rapid advances in the diagnosis, classification, and therapy, the overall survival of lung cancer is still poor and patients’ prognosis remains unfavorable.^[4]^ Though intense research have been used to identify potential molecular prognostic markers for lung cancer, few of them are adopted into clinical use.^[5,6]^ Therefore, new biomarkers with high accuracy for predicting the prognosis in patients with lung cancer are urgently required.

Inactivating somatic mutations of liver kinase B1 (LKB1) are frequently reported in non-small-cell lung cancer (NSCLC), malignant melanoma, and cervical carcinoma.^[7–9]^ However, the results are controversial. LKB1 is a tumor suppressor gene encodes a serine threonine kinase with a stability role in the regulation of cellular metabolism and energy homeostasis.^[10]^ Several studies showed that LKB1 served as a powerful biomarker of tumor functional status could guide clinical trials and patient prognosis assessment.^[11]^ No meta-analysis has been mentioned on LKB1 and its effect on the clinicopathological parameters and prognosis of lung cancer. To address this issue, we performed meta-analysis to comprehensively evaluate the value of LKB1 in patients with lung cancer.

## 2 Materials and methods

### 2.1 Search strategy

The relevant studies were systematically searched with the language restricted to English in the PubMed, Web of Science, Embase, Cochrane library, ClinicalTrials.gov. and Scopus up to September 20, 2020. The search terms included the following keywords:

(“LKB-1” OR “liver kinase B1” OR “STK11” OR “serine-threonine kinase 11”) AND (“lung cancer” OR “lung carcinoma” OR “lung neoplasm” OR “lung tumor”). The references of the review articles and main researches were also searched in order to avoid omission.

### 2.2 Inclusion and exclusion criteria

Studies that were included if they met the following criteria:

1. The pathological diagnosis of lung cancer must be confirmed,
2. the expression of LKB1 in lung tumor tissue was measured by immunohistochemistry (IHC),
3. available data about overall survival (OS), disease-free survival (DFS) and progression-free survival (PFS) that could be accessible,
4. hazard ratio (HR) and 95% confidence interval (CI) of survival data were reported or could be calculated from Kaplan–Meier survival curves,
5. the study was published in English with full text.

The exclusion criteria for this literature were as follows:

1. duplicate publications,
2. laboratory articles, reviews, letters, meta-analysis, reviews, case reports and comments,
3. no mention to LKB1 and lung cancer,
4. lack of information about survival outcomes or survival curves.

### 2.3 Data extraction and quality assessment

The following types of data were extracted from all eligible studies: name of first author, publication year, country, number of cases, gender, smoking, tumour stage, patient's age, follow-up time, cancer histology, cancer type, cutoff value of LKB-1 positivity, detection method of LKB1 expression, survival data (OS, DFS, PFS), HRs, ICs. For some studies from which we could not extract HR and CIs directly, Engauge Digitizer software version 4.1 was used to extract survival rate from Kaplan–Meier curves.^[12]^ Two reviewers independently assessed the quality of the eligible studies using the standard Newcastle-Ottawa Scale (NOS).^[13]^ NOS scores of ≥7 were defined as high quality, 4 to 6 as intermediate quality and 1 to 3 as low quality. All data were cross-checked by two reviewers, and disagreements were resolved by a third researcher.

### 2.4 Statistical analysis

This article was performed using Stata version 12.0 (STATA Corp, College Station, TX) for statistical analysis. Correlation between LKB1 expression and prognosis (PFS, DFS and OS) of patients with lung cancer was evaluated in terms of HRs and 95% CIs. The ORs and 95% CIs were used to evaluate the association between LKB1 expression and clinicopathological characteristics of lung cancer. When it come out a result of Q-test (I^2^ > 50% or *P* < .05) indicated heterogeneity between the studies, the random effects model was used for the meta-analysis. Otherwise, a fixed-effects model was used. Subgroup analysis were carried out to detect sources of heterogeneity. Begg's (rank correlation) and Egger's (regression asymmetry) tests were performed for assessing potential publication bias. Sensitivity analysis was also performed to evaluate the stability of this meta-analysis. The *P* < .05 was regarded as statistically significant.

## 3 Results

### 3.1 Study selection and study characteristics

**Figure 1:**
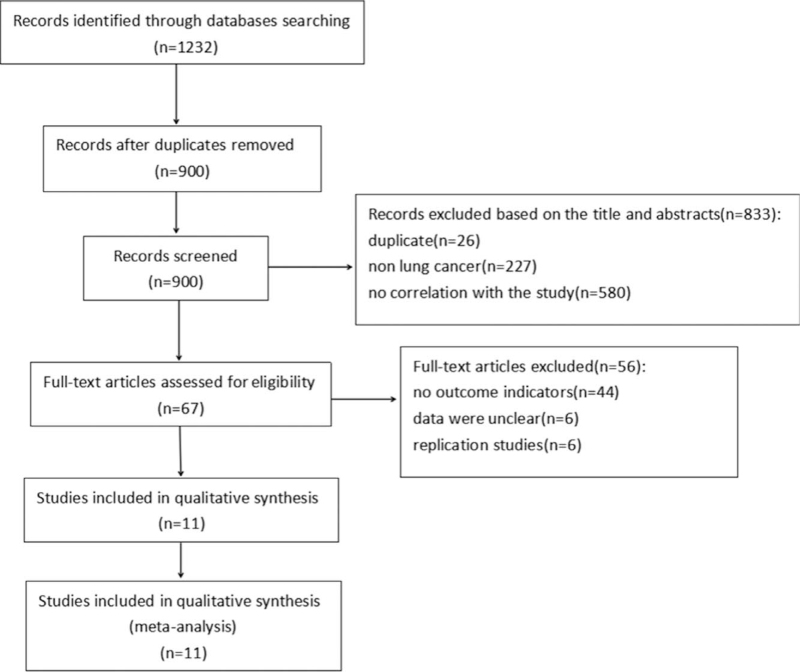
Flow diagram of literature retrieval strategy.

A total of 1232 potentially relevant studies were identified in literature searches. After screening titles and abstracts, a total of 11 studies^[14–24]^ with 1507 patients were included in the meta-analysis, 1221 of which were excluded for reasons are shown in Figure 1. The main characteristics of the eligible studies are summed up in Table 1. Six articles^[15–17,19,22,23]^ originated from China, two are from Korea^[14]^ and Italy^[20]^ and three are from the USA.^[18,21,24]^ Nine articles had statistics on OS,^[14–18,20–23]^ 2 studies had data on DFS,^[23,24]^ and one had data on PFS.^[20]^ The NOS score of included articles ranged from 6 to 8, which suggested that all possessed high methodological quality (Table 2).

**Table 1:** Main characteristics of the eligible studies.

Author	Year	Country	Number	Gender (M/F)	Smoking (never/ever)	Stage	Age (years)/ Medium (range)	Follow-up (months) Medium (range)	Design	Cancer type	Cancer histology	Cutoff value of positive LKB-1	Outcomes	Test method
Ki EH^[14]^	2008	Korea	77	63/14	22/54	I–IV	63 (35–79)	60	Retrospective	NSCLC	ADA, SqCC, Large cell carcinoma, Bronchioloalveolar carcinoma, Small cell carcinoma.	Staining intensity in the tumor > 30%	OS	IHC
Liu SL^[15]^	2013	China	173	91/82	NA	I–II/IIIa–IIIb	N.A.	80	Retrospective	NSCLC	ADA, SqCC	The staining intensity in the tumors matched or exceeded the staining intensity of the normal airway	OS	IHC
Jiang LL^[16]^	2014	China	142	82/60	NA	I–II/III–IV	58.2 (31–84)	31 (3–71)	Retrospective	NSCLC	N.A.	Score ≥ 5	OS	IHC
Tsai LH ^[17]^	2014	China	115	66/49	73/42	I–III	N.A.	>120	Retrospective	Lung adenocarcinoma	ADA	Score > 100	OS	IHC
Calles A^[18]^	2015	USA	126	39/87	21/105	I–IV	N.A.	60	Retrospective	NSCLC	Non-SqCC	Any degree of LKB1staining by IHC was considered positive.	OS	IHC
Li Y^[19]^	2016	China	74	51/23	NA	I–IV	31–83	N.A.	Retrospective	NSCLC	ADA, SqCC	Score ≥ 2	N.A.	IHC
Bonanno L^[20]^	2017	Italy	98	30/68	16/82	N.A.	64 (56–70)	35.4 (6–64.9)	Retrospective	NSCLC	ADA, SqCC, Large cell carcinoma, and Others	Score ≥ 2	OS, PFS	IHC
Muge G^[21]^	2017	USA	305	NA	NA	I–IV	N.A.	100	Retrospective	Lung adenocarcinoma	ADA	N.A.	OS	IHC
Liu MJ^[22]^	2018	China	190	103/87	NA	I–II/III–IV	N.A.	72	Retrospective	Lung adenocarcinoma	ADA	Score ≥ 2	OS	IHC
Qin ZH^[23]^	2019	China	103	76/27	51/52	I–IIIA	N.A.	51.5 (1–64)	Retrospective	NSCLC	N.A.	Score ≥ 4	OS, DFS	IHC
Kyle G^[24]^	2020	USA	104	53/51	15/89	I–III	64 (56.5–73)	89.7 (34.4–135.8)	Retrospective	Lung adenocarcinoma	ADA	Median score	DFS	IHC

ADA = Adenocarcinoma, DFS = disease-free survival, F = female, IHC = immunohistochemistry, LKB1 = liver kinase B1, M = male, N.A. = not available, N = Number of patient, NSCLC = non-small cell lung cancer, OS = overall survival, PFS = progression-free survival, SqCC = squamous cell carcinoma.

**Table 2:** The Newcastle–Ottawa scale (NOS) quality assessment of the eligible studies.

	Selection				Comparability			Outcome	Total
Study	Representativeness of the exposed cohort	Selection of the non-exposed cohort	Ascertainment of exposure	Demonstration that outcome of interest was not present at start of study	Comparability of cohorts on the basis of the design or analysis	Assessment of outcome	Was follow-up long enough for outcomes to occur	Adequacy of follow up of cohorts	quality scores
Ki EH^[14]^	^∗^	^∗^	^∗^	–	–	^∗^	^∗^	^∗^	6
Liu SL^[15]^	^∗^	^∗^	^∗^	–	–	^∗^	^∗^	^∗^	6
Jiang LL^[16]^	^∗^	^∗^	^∗^	–	^∗∗^	^∗^	^∗^	^∗^	8
Tsai LH ^[17]^	^∗^	^∗^	^∗^	–	^∗∗^	^∗^	^∗^	^∗^	8
Calles A^[18]^	^∗^	^∗^	^∗^	–	–	^∗^	^∗^	^∗^	6
Li Y^[19]^	^∗^	^∗^	^∗^	–	–	^∗^	^∗^	^∗^	6
Bonanno L^[20]^	^∗^	^∗^	^∗^	–	–	^∗^	^∗^	^∗^	6
Muge G^[21]^	^∗^	^∗^	^∗^	–	^∗∗^	^∗^	^∗^	^∗^	8
Liu MJ^[22]^	^∗^	^∗^	^∗^	–	^∗∗^	^∗^	^∗^	^∗^	8
Qin ZH^[23]^	^∗^	^∗^	^∗^	–	^∗∗^	^∗^	^∗^	^∗^	8
Kyle G^[24]^	^∗^	^∗^	^∗^	–	^∗∗^	^∗^	^∗^	^∗^	8

-, zero score; ^∗^, one score; ^∗∗^, two scores; a quality score ≥ 6 was considered to be high quality.

### 3.2 Prognostic value of LKB1 over expression for OS in lung cancer

**Figure 2:**
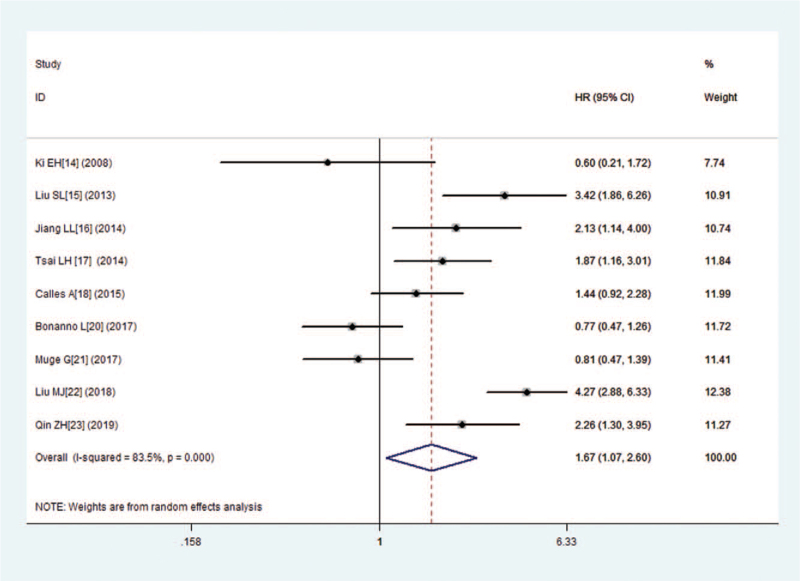
Forest plot of the hazard ratio for the association between the LKB1 and overall survival (OS) in patients with lung cancers.

**Figure 3:**
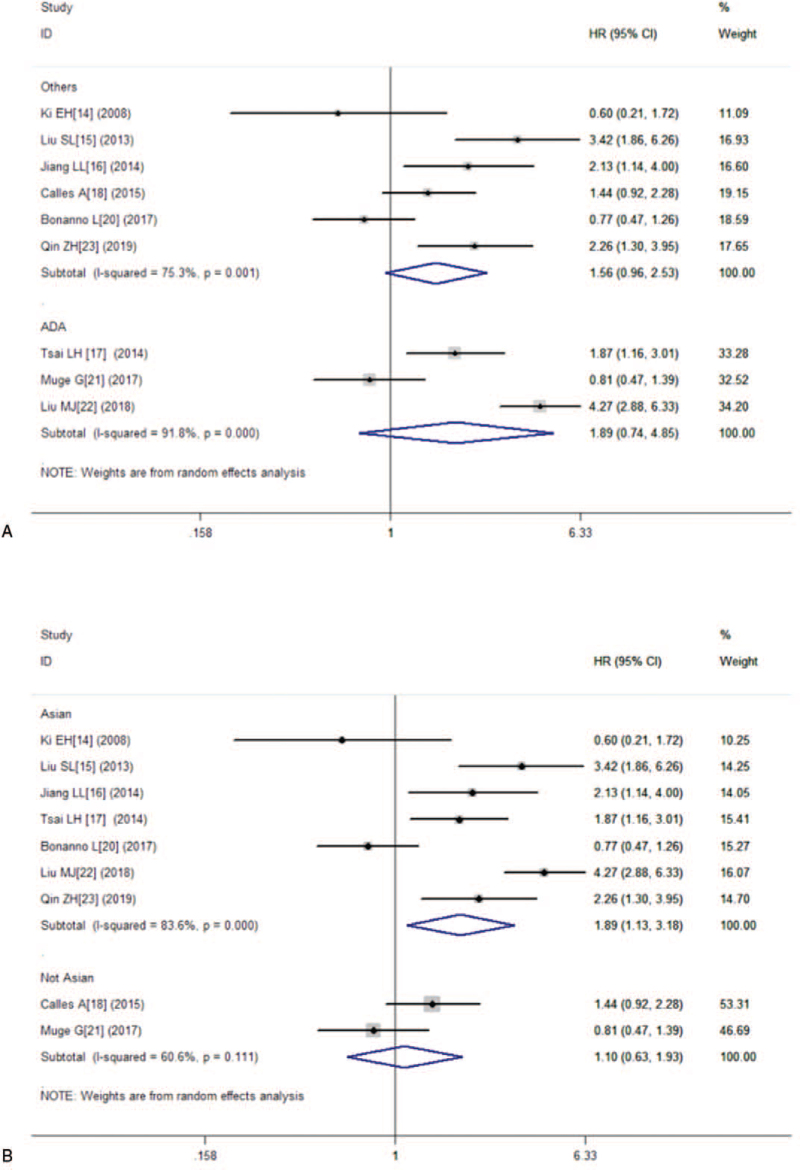
Overall survival (OS) subgroup analyses. (A) OS subgroup analysis in term of different tumor types; (B) OS subgroup analysis of different regions.

Nine studies consisting of 1329 patients reported OS. The combined HR for studies evaluating low expression of LKB1 on OS was 1.67 (95% CI:1.07–2.60, *P* = .024), suggesting that low expression of LKB1 was an indicator of poor prognosis for lung cancer patients (Fig. 2). Because of the significant heterogeneity (I^2^ = 83.5%, *P* = .000), this meta-analysis was calculated by using the random effects model. Furthermore, we performed subgroup analysis on country and cancer type. The results showed that no significant association was found between low expression of LKB1 and OS in lung adenocarcinoma carcinoma (HR = 1.89, 95% CI:0.74–4.85, *P* = .185), either in other types (HR = 1.56, 95% CI:0.96–2.53, *P* = .075) (Fig. 3A). The combined HRs in Asian studies and non-Asian studies were 1.89 (95% CI:1.13–3.18, *P* = .016) and 1.10 (95% CI:0.63–1.93, *P* = .732), respectively (Fig. 3B).

### 3.3 Prognostic value of LKB1 expression for PFS/DFS in lung cancer

**Figure 4:**
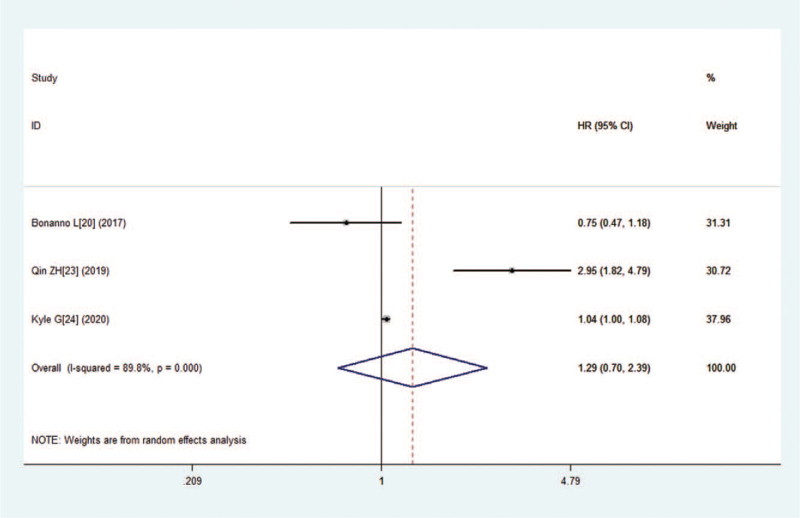
Forest plot of the hazard ratio for the association between the LKB1 and disease-free survival (DFS) and progression-free survival (PFS) in patients with lung cancer.

There were two studies mentioned the data on DFS, and one had data on PFS. This meta-analysis was carried out using the random effects model on account of significant heterogeneity (I^2^ = 89.8%, *P* = .000). The combined HR for studies evaluating low expression of LKB1 on PFS/DFS was 1.29 (95% CI:0.70–2.39, *P* = .410), suggesting that no significant correlation was observed between low expression of LKB1 and PFS/DFS (Fig. 4).

### 3.4 High LKB1 expression and clinicopathological characteristics in lung cancer

To systematically analyzed the role of LKB1 expression as a biomarker in lung cancer, we explored the correlation between low expression of LKB1 and clinicopathological characteristics. A total of 7 studies described the association between LKB1 expression and clinicopathological factors, including age, gender, histological differentiation, nodal metastasis, smoking, tumor stage, histopathological stage (Table 3). Low expression of LKB1 was association with histological differentiation (poor vs. moderate or well, OR = 4.135, 95% CI:2.524–6.774, *P* = .000), nodal metastasis (absent vs present, OR = 0.503, 95% CI:0.303–0.835, *P* = .008), smoking (yes vs no, OR = 1.765, 95% CI:1.120–2.782, *P* = .014). However, LKB1 expression had no significant association with age (<60 vs ≥60, OR = 1.073, 95% CI:0.639–1.800, *P* = .790), gender (male vs. female, OR = 0.997, 95% CI: 0.756–1.314, *P* = .981), histopathological stage (I-II vs III-IV, OR = 0.814, 95% CI: 0.596–1.112, *P* = .196), tumor stage (T1-T2 vs T3-T4, OR = 0.729, 95% CI: 0.262–2.029, *P* = .545) (Table 3).

**Table 3:** Meta-analysis of reported clinicopathological characteristics in the included studies.

				Test for heterogeneity		
Parameters	Number of studies	OR (95%CI)	*P* value	I^2^(%)	*P*	Statistic model
Age (<60 vs ≥60)	4	1.073 (0.639–1.800)	.790	50.20	.110	Random
Gender (male vs female)	8	0.997 (0.756–1.314)	.981	33.40	.161	Fixed
Smoking (yes vs no)	4	1.765 (1.120–2.782)	.014	49.70	.113	Fixed
Histological differentiation (poor vs moderate or well)	3	0.814 (0.596–1.112)	.196	0.00	.712	Fixed
Nodal metastasis (absent vs present)	7	0.503 (0.303–0.835)	.008	63.00	.013	Random
Histopathological stage (I-II vs III-IV)	7	0.814 (0.596–1.112)	.196	47.40	.007	Random
Tumor stage (T1-T2 vs T3-T4)	4	0.729 (0.262–2.029)	.545	76.80	.005	Random

### 3.5 Sensitivity analysis

**Figure 5:**
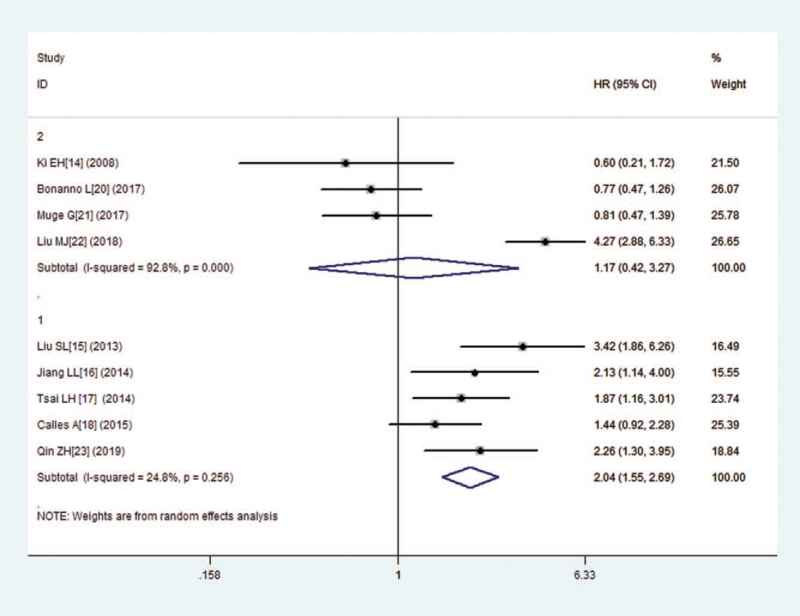
Sensitivity analysis of the association between LKB1and overall survival.

Sensitivity analysis was used to explore the potential heterogeneity within the eligible studies of OS analysis (Fig. 5). Each of the articles were successively excluded to judge the robustness of the pooled results. However, the results shown that were significant heterogeneity. According to the OS analysis, the heterogeneity test found no significant heterogeneity after excluding four studies^[14,20–22]^ (I^2^ = 24.8%, *P* = .256). The pooled HR for OS in patients with high versus low expression of LKB1was 2.044 (95% CI: 1.551–2.694, *P* = .000), suggesting a poor prognostic role of LKB1 expression. Therefore, we must be careful in drawing a conclusion regarding with OS.

### 3.6 Publication bias

**Figure 6:**
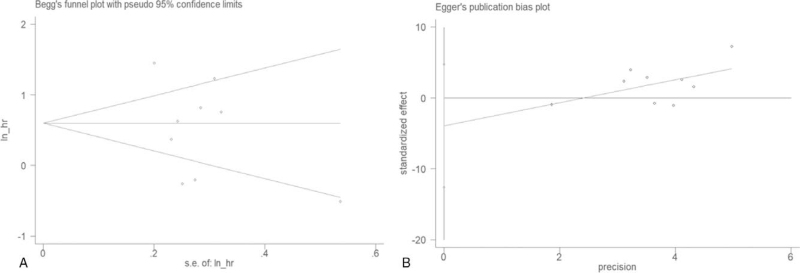
Funnel plots for detecting publication bias in terms of survival data. (A) Begg's funnel plot using data of overall survival to detect publication bias; (B) Egger's funnel plot using data of overall survival to detect publication bias.

A potential publication bias was detected by Begg's test and Egger's test. Our findings with Begg's test (p = 0.917) and Egger's test (p = 0.318) implied no publication bias (Fig. 6).

## 4 Discussion

The cancer suppressor LKB1 is an essential serine/threonine kinase, which induces multifarious cellular processes such as cell metabolism, cell proliferation and cell migration.^[25]^ Somatic mutations or loss-of-function alterations of LKB1 were found in different tumor types, such as cervical carcinoma, breast cancer, pancreatic cancer and non-small-cell lung cancer (NSCLC).^[26–30]^ What's more, LKB1 is the most commonly mutated genes in NSCLC and approximately 30–35% of lung adenocarcinomas loss of the function occurring.^[31]^ Though emergence of chemotherapy immunotherapy and targeted therapy are developing, lung cancer still a huge threat for human health due to the drug resistance and metastasis. A previous study has provided that LKB1 loss triggers complex changes in tumor microenvironment, suggesting a potential role in the response to anti-angiogenic treatment.^[32]^ A number of articles have reported the prognostic value of LKB1 expression in tumors among patients with lung cancer and the results remain controversial. Thus, it is urgent to seek available biomarkers for early tumors detection and prognosis evaluation.

Recently, more and more attention is focused on immunotherapy targets in treating lung cancer, which have showed promising outcomes. LKB1 was deemed to be a new biomarkers in immunological therapy with the growing recognition of LKB1and its metabolic pathways.^[33]^ The researches have showed that LKB1 directly phosphorylates and activates AMPK, which works as a master sensor of cellular growth and proliferation.^[34,35]^ A novel set of findings were presented which remind that not only oncogene driver mutations but also tumor-suppressor gene mutations can modify the immune microenvironment in lung cancer.^[36]^ Furthermore, the data have indicated that LKB1 mutation in NSCLC conferred enhanced radio sensitization in combination with trametinib, suggesting LKB1 mutation as a biomarker for patient's trametinib and radiotherapy combination therapy.^[37]^ A previous study has provided certain information regarding the prognostic value of LKB1 in patients with solid tumours.^[38,39]^ However, no meta-analysis have been performed to evaluate the prognostic value of LKB1 expression in lung cancer. This meta-analysis is aimed to investigate the effect of LKB1 expression on the prognosis and clinicopathological characteristics in lung cancer.

This meta-analysis included 11 eligible articles with a total of 1507 patients. We found that low expression of LKB1 may be an indicator of poor prognosis for lung cancer patients. Furthermore, we performed subgroup analysis on country and cancer type. The results showed that no significant association was found between low expression of LKB1 expression and OS in lung adenocarcinoma carcinoma and other types, in Asian studies and non-Asian studies. Our results showed that there were no association between low expression of LKB1 and DFS/PFS. Concerning clinicopathologic factors, low expression of LKB1 was associated with histological differentiation, nodal metastasis, and smoking. However, LKB1 expression had no significant association with age, gender, histopathological stage, and tumor stage.

The results of our meta-analysis should be interpreted with caution given several limitations. First, all included studies were published in the English language which may lead to publication bias. Secondly, although the Begg's test and Egger's tests revealed no publication bias, most eligible articles were from Asia, which may lead to publication bias. Thirdly, sensitivity analyses revealed that the correlation between LKB1 over expression and OS was unstable, which might be explained by the small sample sizes. Therefore, we must be careful in drawing a conclusion regarding the prognostic significance of LKB1 in lung cancer.

## 5 Conclusions

In conclusion, this meta-analysis suggested that low expression of LKB1 may predict unfavorable prognosis, worse histological differentiation and earlier nodal metastasis of lung cancer. Furthermore, high quality and multicenter studies should be carried out to clarify the effect of LKB1 expression in lung cancer.

## Author contributions

**Conceptualization:** Chenggong Wei.

**Data curation:** Chunxuan Lin, Xiaochun Lin, Kunpeng Lin, Jialiang Tan.

**Formal analysis:** Chunxuan Lin, Xiaochun Lin, Kunpeng Lin.

**Investigation:** Chunxuan Lin, Xiaochun Lin, Kunpeng Lin.

**Methodology:** Jialiang Tan, Taisheng Liu.

**Project administration:** Taisheng Liu, Chenggong Wei.

**Software:** Chunxuan Lin.

**Supervision:** Taisheng Liu.

**Writing – original draft:** Chunxuan Lin, Xiaochun Lin, Kunpeng Lin.

**Writing – review & editing:** Taisheng Liu, Chenggong Wei.
